# Supplementary material for: Expanded genome and proteome reallocation in a novel, robust Bacillus coagulans strain capable of utilizing pentose and hexose sugars
Source: mSystems. 2024 Oct 8;9(11):e00952-24. doi: 10.1128/msystems.00952-24 (PMC11575207; doi:10.1128/msystems.00952-24)
Supplement: Supplemental Material — Figures S1-S5. [file msystems.00952-24-s0005.pdf]

Supplementary Materials

Expanded Genome and Proteome Reallocation in A Novel, Robust *Bacillus coagulans* Capable of Utilizing Pentose and Hexose Sugars

David Dooley[1], Seunghyun Ryu[1], Edwards Jackson[2], Richard Giannone[3], Bruce Dien[2], Cong T. Trinh[1,*]

[1]Department of Chemical and Biomolecular Engineering, University of Tennessee Knoxville

[2]Bioenergy Research Unit, The National Center for Agricultural Utilization Research, USDA-ARS, Peoria, IL 61604

[3]Biosciences Division, Oak Ridge National Laboratory, Oak Ridge, TN 37831

*Corresponding author. Email: ctrinh@utk.edu

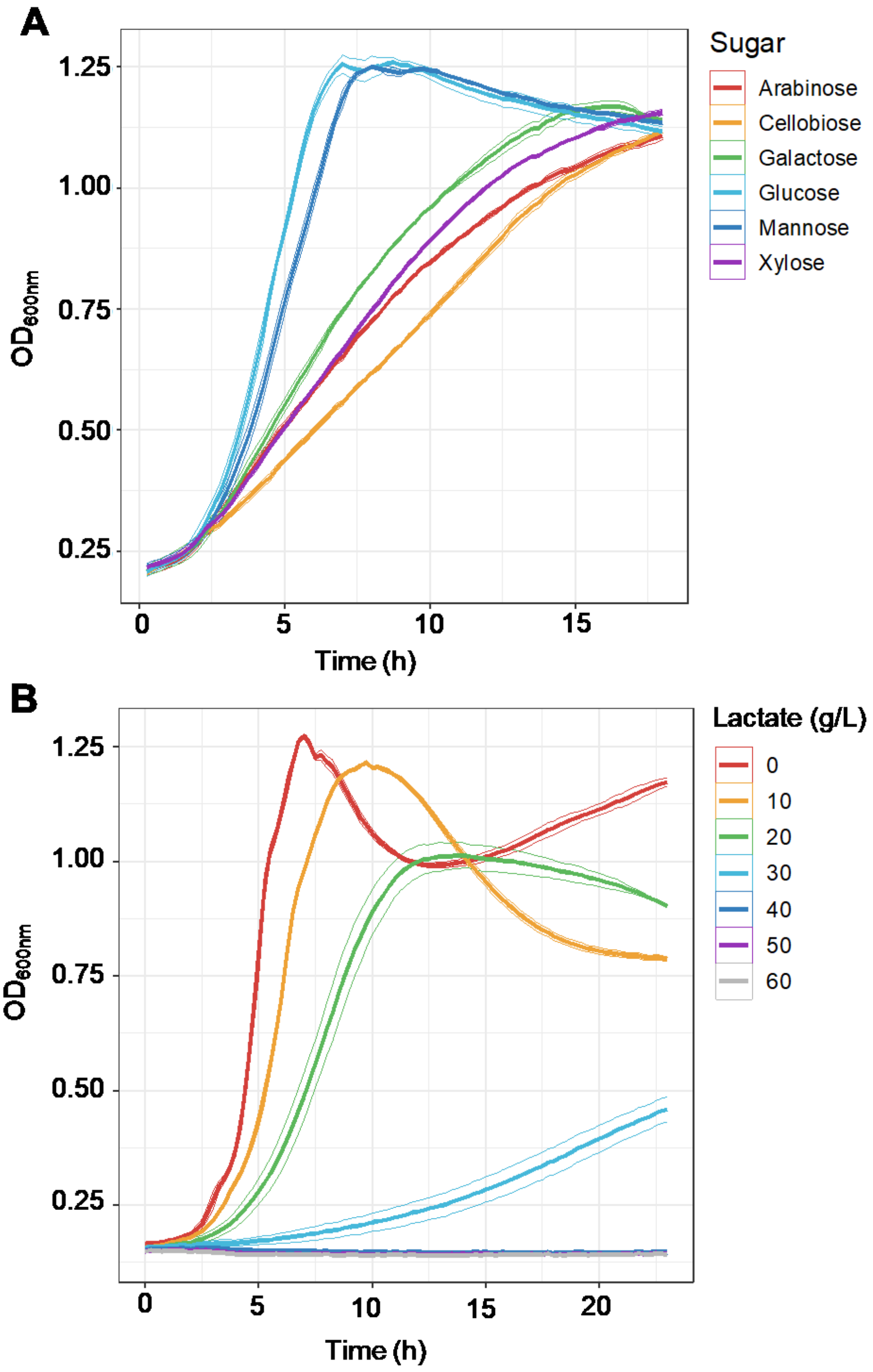

Figure S1: Growth kinetics of *B. coagulans* B-768 growing **(A)** on various C5 and C6 sugars and **(B)** in presence of various concentrations of lactate at 50°C.

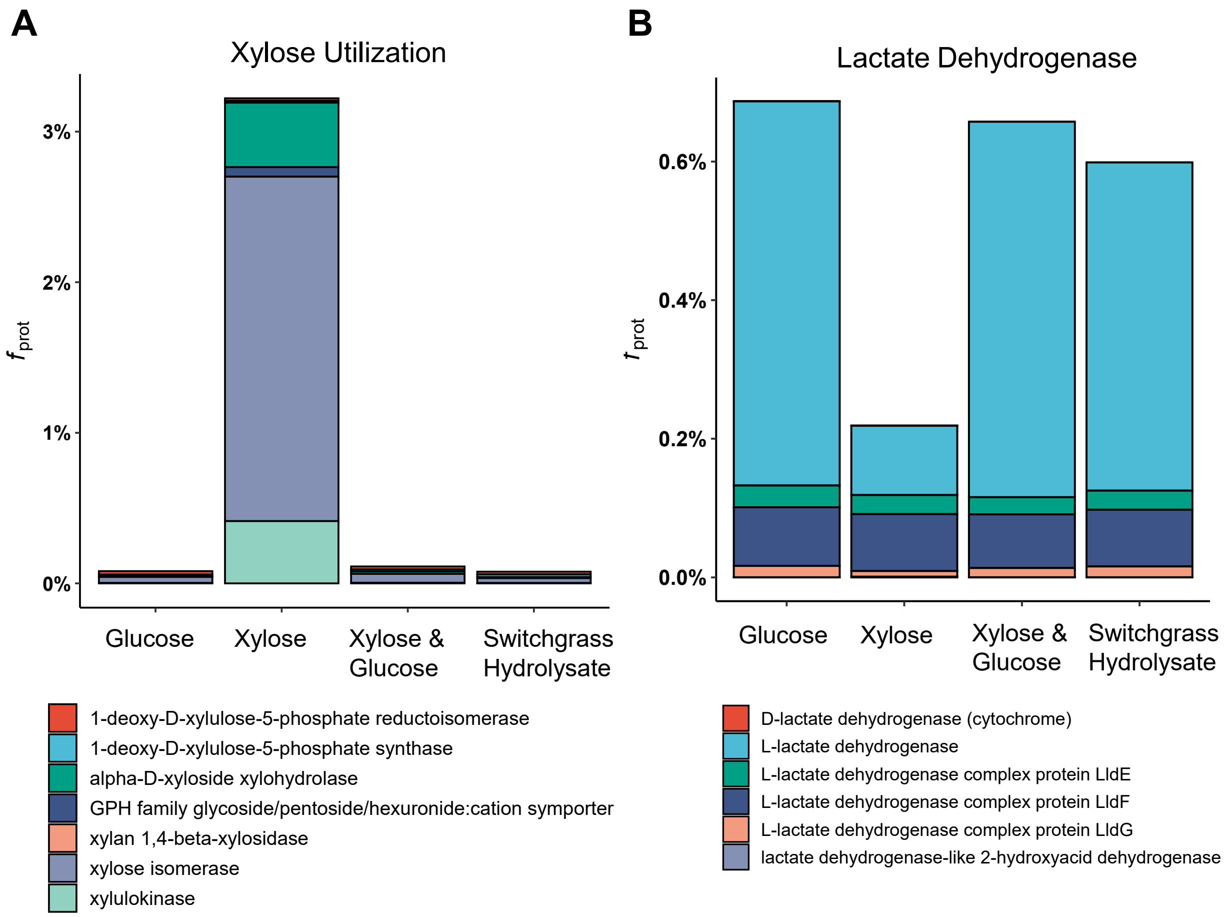

Figure S2: Proteome reallocation for **(A)** xylose utilization and **(B)** lactate biosynthesis of *B. coagulans* B-768 growing xylose, glucose, a mixture of glucose and xylose, and switchgrass hydrolysate (SGH).

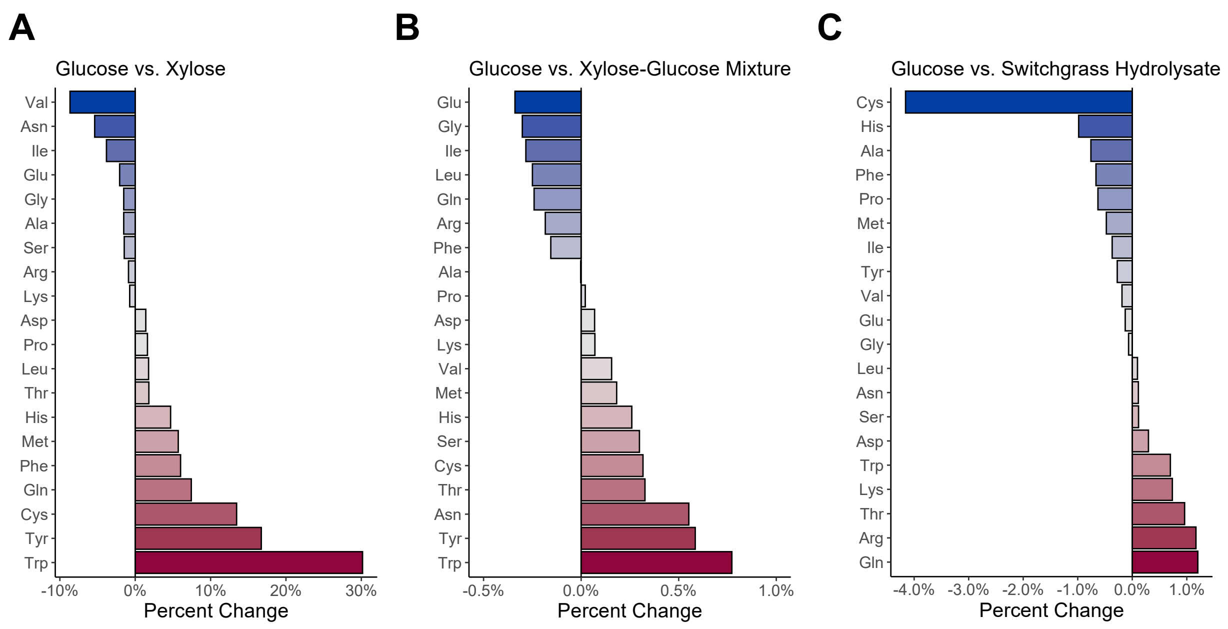

Figure S3: Percent of change in amino acid reallocation in measured proteomes of *B. coagulans* B-768 growing on various sugars including **(A)** glucose versus xylose, **(B)** glucose versus a mixture of glucose and xylose, and **(C)** glucose versus switchgrass hydrolysate (SGH).

Differential Expression of Xylose vs. Glucose

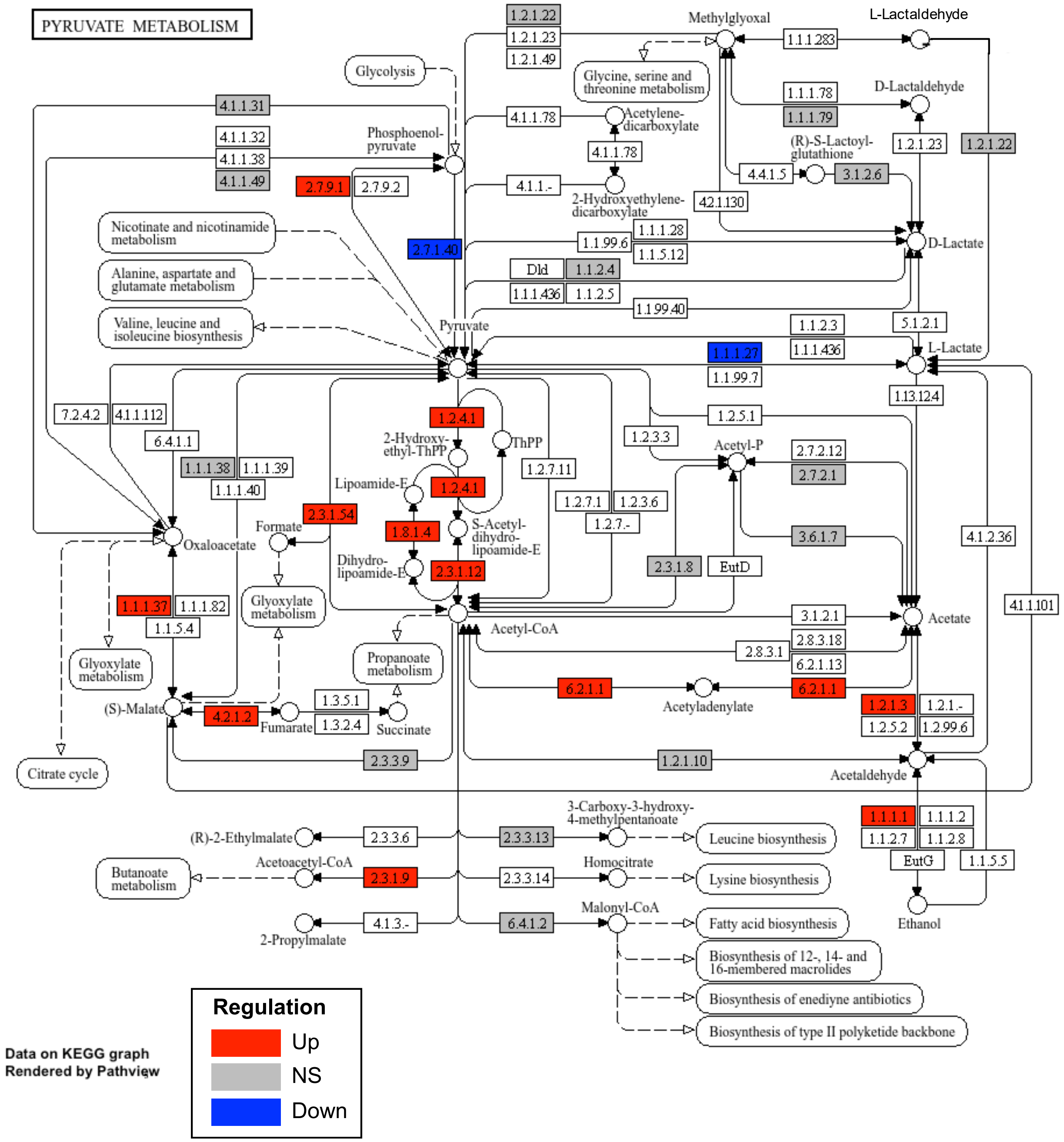

Figure S4: Differential expression of proteins involved in Pyruvate Metabolism of *B. coagulans* B-768 growing on xylose and glucose. “Up” regulation, adjusted p-value < 0.05 and log2 fold change > 1; “NS” = Not Significant” “Down” regulation, adjusted p-value < 0.05 and log2 fold change < -1.

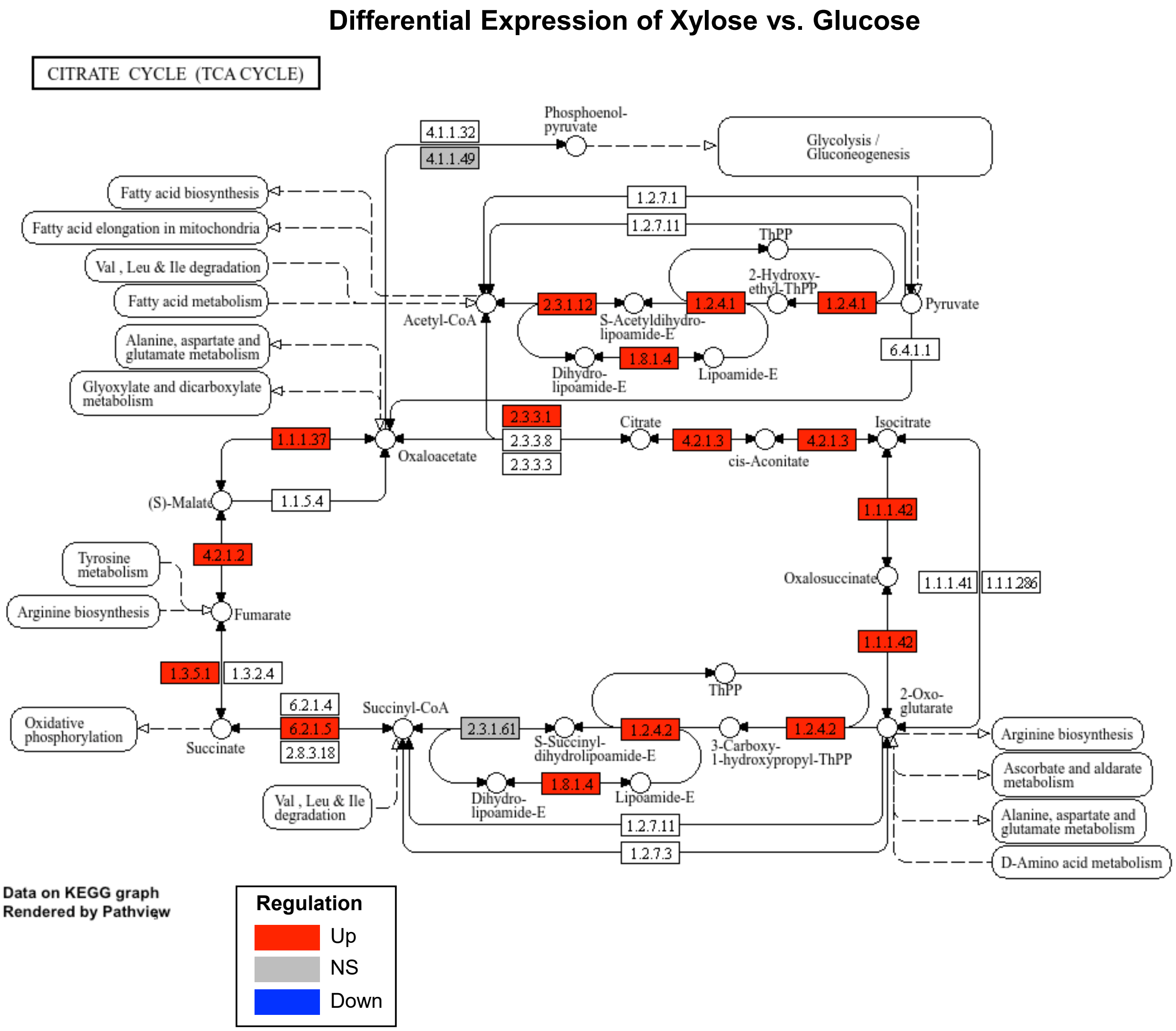

Figure S5: Differential expression of proteins involved in TCA cycle of *B. coagulans* B-768 growing on xylose and glucose. “Up” regulation, adjusted p-value < 0.05 and log2 fold change > 1; “NS” = Not Significant” “Down” regulation, adjusted p-value < 0.05 and log2 fold change < -1.
